# Efficient electrochemical remediation of microcystin-LR in tap water using designer TiO_2_@carbon electrodes

**DOI:** 10.1038/srep41326

**Published:** 2017-02-01

**Authors:** Germán Sanz Lobón, Alfonso Yepez, Luane Ferreira Garcia, Ruiter Lima Morais, Boniek Gontijo Vaz, Veronica Vale Carvalho, Gisele Augusto Rodrigues de Oliveira, Rafael Luque, Eric de Souza Gil

**Affiliations:** 1 Faculdade de Farmácia, Universidade Federal de Goiás, Goiânia, Brazil; 2 Departamento de Química Orgánica, Universidad de Cordoba, Spain; 3 Instituto de Química, Universidade Federal de Goiás, Goiânia, Brazil

## Abstract

Microcystin-leucine arginine (MC-LR) is the most abundant and toxic secondary metabolite produced by freshwater cyanobacteria. This toxin has a high potential hazard health due to potential interactions with liver, kidney and the nervous system. The aim of this work was the design of a simple and environmentally friendly electrochemical system based on highly efficient nanostructured electrodes for the removal of MC-LR in tap water. Titania nanoparticles were deposited on carbon (graphite) under a simple and efficient microwave assisted approach for the design of the electrode, further utilized in the electrochemical remediation assays. Parameters including the applied voltage, time of removal and pH (natural tap water or alkaline condition) were investigated in the process, with results pointing to a high removal efficiency for MC-LR (60% in tap water and 90% in alkaline media experiments, under optimized conditions).

Water is the utmost essential natural resource, being mandatory for all living species. Water quality is consequently a milestone for public health, disease control and sustainability and prioritaire in terms of research and development programs^1^,^2^,^3^. Population growth as well as globalization over past decades have remarkably increased waste generation as well as the environmental introduction of several emerging pollutants^4^. Emerging contaminants are generally defined as any chemical or microorganism that is not commonly monitored in water but may be a candidate for future regulation/s depending on (eco)toxicity, potential human health effects, public perception, and frequency of occurrence in environmental media^5^. Immune toxicity, neurotoxicity, endocrine disruption, and carcinogenicity are among health consequences of exposure to these contaminants^4^.

The myriad of emerging chemical pollutants includes multi-class compounds, mainly originated from anthropogenic activity, *i.e.* pharmaceuticals, cosmetics, sweeteners, pesticides, nanomaterials, plasticizers and flame retardants^3^,^4^,^6^,^7^. Natural toxins are also representative biological sources of emerging pollutants of significant concern^8^,^9^,^10^. In this context, the hepatotoxic and/or neurotoxic cyanotoxins upsurge in hydric resources as one of the major threats for water quality^11^, with microcystins being most widespread in fresh waters^12^,^13^. Chemically, microcystins are cyclic heptapeptides composed by ubiquitous L-amino acids (alanine, methionine, aspartic acid and glutamine) as well as the unique β-amino acid (ADDA). Microcystin-LR has been identified as one of the toxins of highest priority, in which the variable standard L-amino acids, leucine and arginine complete the cycle (Fig. 1). In 1998, the World Health Organization stressed the potential public health importance of the occurrence of microcystin in drinking water and adopted a provisional guideline value for microcystin-LR of 1.0 μg/L^2^,^10^,^14^.

Due to the high toxicity of MC-LR, highly efficient remediation methods have been under development in recent years, with advanced oxidation processes focused on mineralization as major target^15^,^16^,^17^,^18^,^19^. Electrochemical remediation arose as a interesting alternative with few reported protocols to date which include a catalytic electrodes with Ti, Ir, Pt, BDD^20^,^21^,^22^,^23^. Although these electrodes indeed show a high efficiency in electrochemical remediation (above 90% under optimum conditions), they generally have higher costs as compared to metallic electrodes^24^, while Cu, Fe or Zn electrodes can generate others water contaminants (e.g. copper oxides).

Based on these premises, the proposed approach was aimed to the design of a cheap, highly efficient and environmentally friendly electrochemical alternative to degrade MC-LR in drinkable water using TiO_2_@C electrodes. The proposed electrochemical removal employs electrodes made from pencil and carbon sticks from recycled batteries.

The pencil graphite used as anode was modified with TiO_2_ using a previously reported microwave-assisted technique^22^ in order to achieve supported titania nanoparticles on the carbonaceous supports with enhanced electrocatalytic properties.

The graphite source utilised combined with TiO_2_ allowed for a high efficiency towards low cost and more environmentally friendly unprecedented electrodes for the proposed electrochemical remediation. The efficiency of synthesized TiO_2_@C anodes was compared to that of unmodified graphite and titanium wires focusing on the degradation of MC-LR and the formation of degradation products.

## Results and Discussion

### Anode characterization

**Figure 2:**
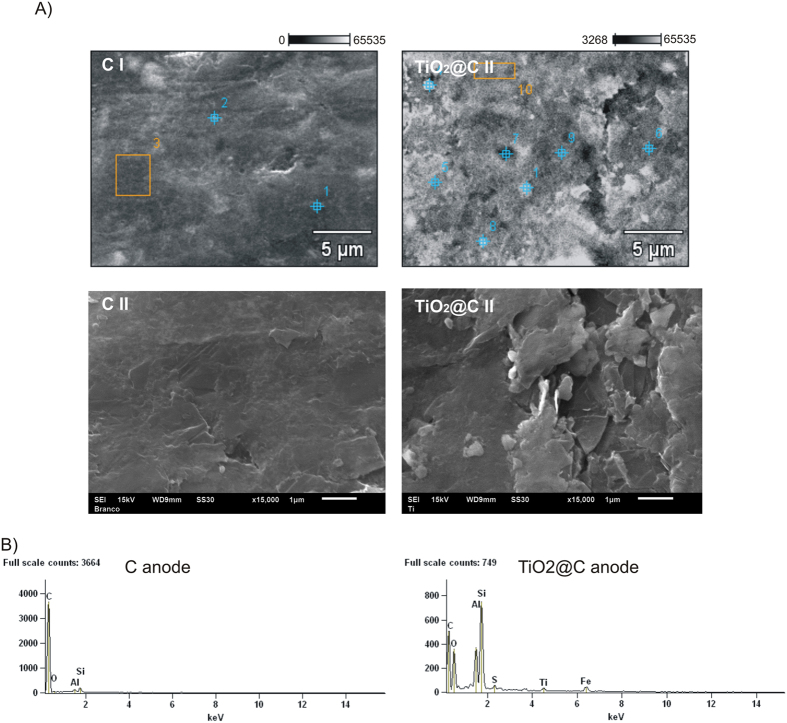
SEM and DRX characterization. (**A**) SEM images at magnification of 5.000 (I) and 15.000 (II), the numbers represents the points where were done EDX analyses; (**B**) EDX spectra obtained for C and TiO_2_@C anodes at the points 1 and 7 respectively.

SEM and DRX characterization for C and TiO_2_@C anodes are depicted in Fig. 2. SEM images at high magnification seemed to point to the presence of titania nanoparticles supported on the smooth carbon surface (Fig. 2A), TiO_2_@C I) as compared to the parent carbonaceous material (Fig. 2A), C I).

**Figure 3:**
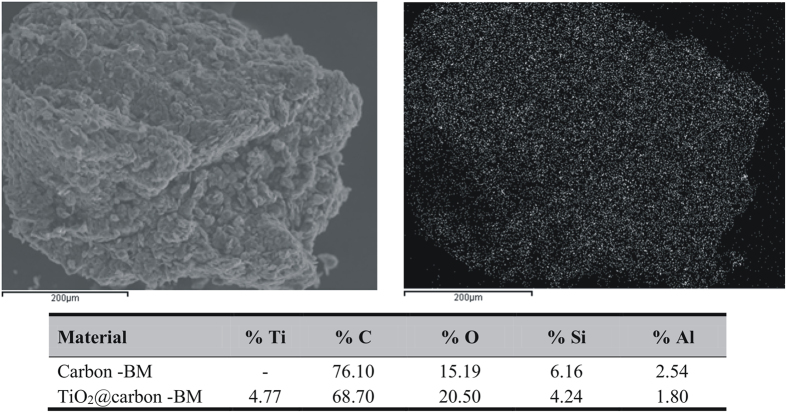
SEM and elemental mapping. Distribution of TiO_2_@C (right image) depicting nice and homogeneously distributed Ti in the material (white dots on a black background). Table with C I and TiO_2_@C I elementary composition.

These findings were in good agreement with mapping and EDX results of both materials (several points were analyzed from Fig. 2B)- C I and TiO_2_@C I and compared for an average value in which Ti could be detected in relatively low loadings (4.77%) and very homogeneously distributed in the material (Fig. 3, right image). The commercial graphite material contained also relevant quantities of Si and Al in its composition (Fig. 2B).

### Electrochemical activity and degradation assay

**Figure 4:**
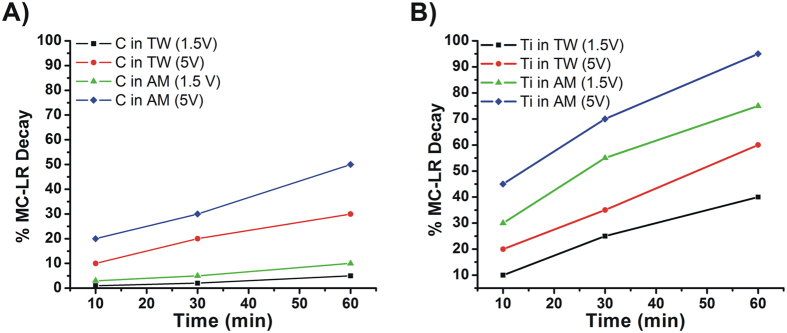
Electrochemical remediation of MC-LR in TW and AM with commercial electrodes. Was used C (**A**) and Ti (**B**) anodes at different applied voltages (a Pt wire was used as cathode in both cases).

Upon characterization, the electrochemical activity of the electrodes was tested in the degradation of MC-LR. Preliminary experiments were conducted with a C cylinder as anode and a platinum wire as cathode in tap water (TW) and alkaline media (AM). An almost negligible MC-LR degradation could be observed for the TW solution at 1.5 V (only slightly superior to the estimated standard deviation RSD% after 1 h). Higher voltage (5 V) experiments improved MC-LR degradation efficiency in TW to two-fold. Comparatively, MC-LR removal significantly improved in AM solutions, which can be due to chemical hydrolysis (Fig. 4A).

The use of a commercial pure Ti wire as anode system remarkably improved the degradation kinetics of MC-LR (Fig. 4B), reaching a maximum of ca. 50% degradation for TW and over 90% for AM, respectively, at high voltages (5 V). MC-LR degradation at the metallic electrode might occur directly by electrochemical oxidation or indirectly due to OH^•^ generation.

**Figure 5:**
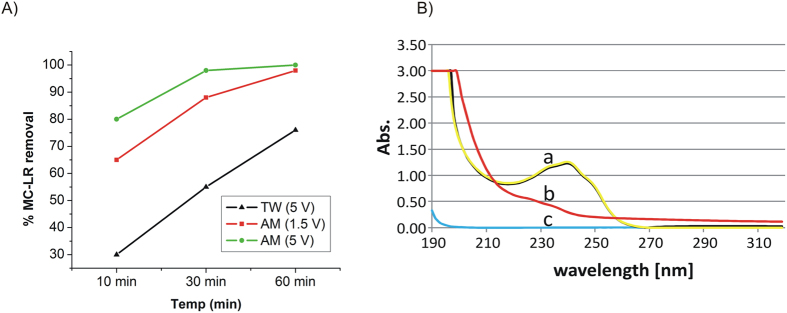
MC-LR remediation by TiO_2_@C. Electrochemical removal of MC-LR by using TiO_2_@C anode (**A**) and UV spectra (**B**) for MC-LR solution (a), MC-LR solution after 60 minutes electrolysis at TiO_2_@C anode in TW condition (b) and pure TW (c).

Interestingly, the use of the designed TiO_2_@C anode significantly predated results from commercially pure Ti wire despite the low titania content (<5%), exhibiting an optimum performance for MC-LR degradation as depicted on Fig. 5. An almost complete mineralization (>90% MC-LR electrochemical degradation) was observed for AM at 1.5 V after 60 minutes of electrolysis (Fig. 5A). Most importantly, a remarkable 60% degradation was obtained for TW samples (low conductivity for electrochemical processes) at 5 V, with respect to a 45–50% for the commercial pure Ti wire (Fig. 5B).

Given that the oxygen evolution potential is ca.1,7 V^25^, the experimental conditions were optimized for 1.5 V to minimise water oxidation. Figure 5A show that MC-LR removal was in fact very similar at the two selected reaction conditions (1.5 and 5 V) under AM solution (60 min) using the designed TiO2@C anode.

The observed improvement of MC-LR removal in AM solution is due to the addition of sodium carbonate and related with the increase of conductivity (200 S.cm^−2^ in TW as compared to 4008 S.cm^−2^ in AM), allowing higher current flow with reduced energy consumption, in good agreement with previous literature reports^23^. Electroflotation processes were not observed during the different electrochemical degradation tests, previously reported using iron and aluminum anodes^26^.

The energy efficiency of the different treatments can be compared using the proposed electric power equation (1) and the time of reaction as:


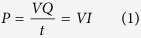


where: P is electric power; Q is electric charge in coulombs; t is time in seconds; I is electric current in amperes; and V is electric potential or voltage in volts.

The results presented in Fig. 5A indicate a high removal efficiency of the designed TiO2@C anode at only 1.5 V (above 80%), which may correspond with 0,0025 W of energetic consumption, instead of 0,042 W required for the commercial Ti anode employed for comparative purposes in this work (see Fig. 4B) to reach a similar efficiency.

**Figure 6:**
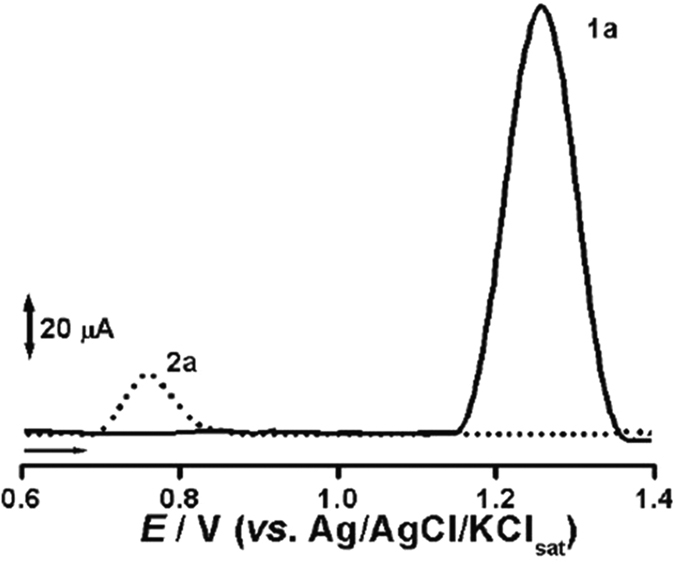
Electro-degradation activities of the designed TiO_2_@C DPV. DP voltammograms obtained for MC-LR before (^**__**^) and after anodic treatment (•••) at TiO_2_@C in TW for 30 minutes and applied voltage of 5 V, both in 0.1 M pH 7.0 PBS at glassy carbon electrode. Scan rate of 10 mV s^−1^, pulse amplitude of 50 mV.

The excelling MC-LR electro-degradation activities of the designed TiO_2_@C anode was also evidenced by DPV, in which the disappearance of anodic peak 1a can be clearly noticed, *E*_p1a_ = 1.25 V after 30 minutes of electrolysis (Fig. 6). The appearance of peak 2a, can be attributed to electrochemically generated hydroxylated products during the oxidative process.

### MC-LR degradation analyses

**Figure 7:**
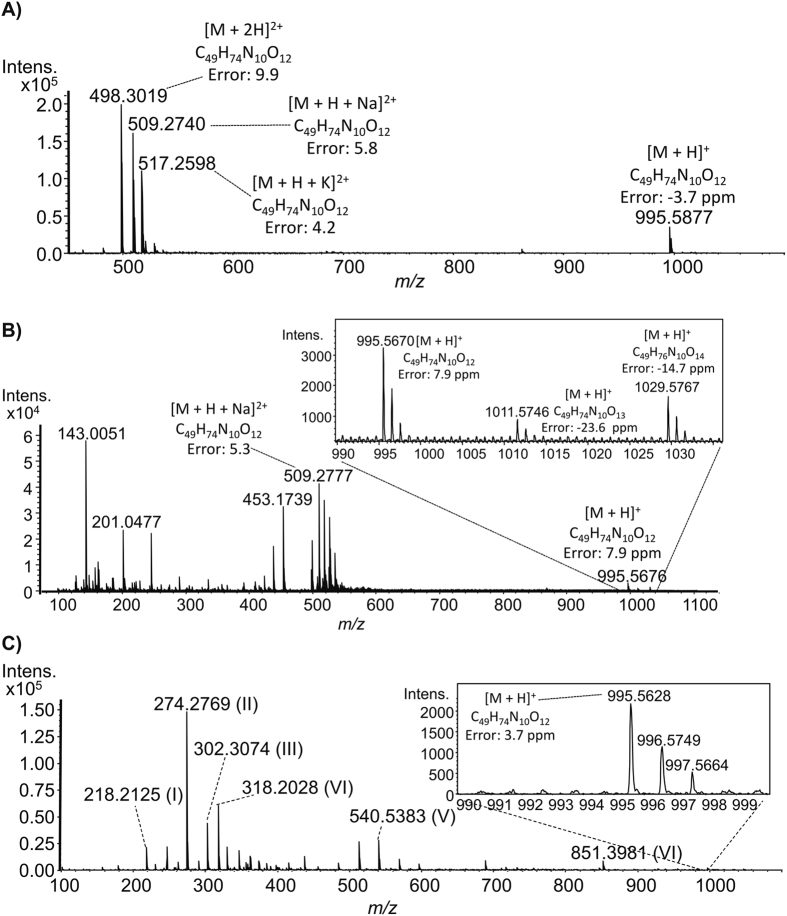
MC-LR degradation analyses by mass spectrometry. ESI(+) Q-TOF mass spectra of standard unconverted Microcystin-LR (**A**), pure Ti wire anode treatment (**B**), and TiO_2_@C anode treatment (**C**). All figures was imported-exported with 600 dpi using CorelDraw 12.0.0.458 but none was edited. Graphics were done with OriginPro 8 SRO. After, they were imported-exported with 600 dpi using CorelDraw 12.0.0.458.

The analysis of degradation products was subsequently conducted by ESI( + )Q-TOF mass spectrometry in order to investigate the identity of the degradation products derived from the electrochemical degradation of MC-LR. Figure 7 shows the Q-TOF mass spectra of standard unconverted MC-LR as compared to pure Ti wire anode and TiO_2_@C anode treated solutions.

MC-LR mass spectra revealed that microcystin was detected as single and double-charged ions: [M + H]^+^ of *m/z* 995.5877; [M + H + K]^2+^ of *m/z* 517.2598; [M + H + Na]^2+^ of *m/z* 509.2740; and [M + 2 H]^2+^of *m/z* 498.3019 respectively (Fig. 7A). Figure 7B illustrates the mass spectrum of TiO_2_@C anode treatment. MC-LR ([M + H]^+^ of *m/z* 995.5877) was detected in lower abundance with respect to the standard unconverted MC-LR solution. Two degradation products, *m/z* 1011.5746 and 1029.5767, could be detected as reaction intermediates identified as oxidation products of MC-LR, in good agreement with previous studies^27^,^28^,^29^. Most importantly, TiO_2_@C anode treatment mass spectra (Fig. 7C) illustrates a significant reduction of MC-LR abundance intensity in addition to the detection of six degradation products (I-VI, structures shown in Table 1). Interestingly, most degradation products detected in this work differ from previous studies, probably due to the experimental conditions^30^.

Major oxidation products detected for MC-LR degradation by commercial pure Ti wire anode treatment could not be detected in the case of TiO_2_@C anode treatment. We believe that the more intense degradation observed in the case of the latter electrode could be the main reason (as pointed out by UV-Vis, DPV and MS). These facts can be based on two points: i) the degradation of MC-LR is promoted by a hydroxyl radical-based mechanism that led to oxidation products, fully mineralised priort to MS analyses; or ii) an alternative degradation mechanism of MC-LR may take place under the investigated conditions different from the initial oxidation of MC-LR.

## Conclusions

The removal of microcystins and cyanobacteria in fresh water suppliers under electrochemical conditions was achieved in this work by designed highly efficient electrodes. A nanostructured TiO2@C exhibited enhanced electrochemical activity as compared to commercial pure Ti anodes. The high removal rate is in principle consistent with electro-generation of reactive species and with literature reports for similar types of electrochemical remediation using other anodes. Besides, the recognized electrocatalytical properties of TiO, the increment of electroactive surface of nano-systems and the increase of water conductivity allowing larger current under identical voltage promoted the direct and indirect electrooxidation of MC-LR. In turn, deprotonating alkaline medium may contribute to both chemical hydrolysis and electrochemical oxidation. The high efficiency mass spectrometry allowed the identification of six degradation products of MC-LR, whose quantities accordingly vary to the anode used and electrolytic conditions.

## Materials and Methods

### Reagents

Microcystin-LR (MC-LR) ethanol solutions (500 μg/500 μL) were purchased from Cayman Chemical Company (Ann Arbor, Michigan, USA).

Chemicals used for electrode modification, remediation assays, all of ACS grade, were purchased from Sigma-Aldrich and used without any further purification. MS and HPLC grade solvents were purchased from J.T. Baker.

### Synthesis and characterization of electrodes

#### Commercial anodes

The Ti wire (∅ 2 mm) was purchased from Realum (São Paulo, SP, Brazil) as compared to a C cylinder (∅ 2 mm), commonly used for drawing purposes, purchased from a local stationery. Such commercial materials were cut in order to reach 3 cm length and then used as anodes without any prior treatment.

#### TiO_2_@Carbon anode

The aforementioned C cylinder (3 cm x ∅ 2 mm; c.a 1.5 g) was modified with nanosized titania nanoparticles, supported via microwave deposition of Titanium (IV) isopropoxide at 5%^31^. Briefly, the C cylinder was immersed in a solution of 1 mL of Titanium (IV) isopropoxide and 3.5 mL ethanol. The Microwave deposition was carried out in a CEM-Discover model with PC control. Experiments were conducted in a closed vessel under continuous stirring and without cooling. The method was temperature controlled at 120 °C, power irradiation and pressure were set at 220 W and 220 PSI respectively in line with previously optimized reports^32^,^33^.

Scanning Electron Microscopy (SEM) experiments for electrode caracterization were conducted in a JEOL Ltd. (Musashino, Akishima, Tokyo, JAPAN) JSM-6610 model. The magnification range was 1.000 to 15.000, with an accelerating voltage of 15.0 kV. The elemental analysis of electrodes was performed by means of energy-dispersive X-ray spectroscopy (EDS).

### MC-LR sample preparation

To evaluate the efficiency of modified electrodes on the MC-LR electrochemical oxidation, two sample solutions were prepared. The first was prepared by dissolution of the content of one entire MC-LR vial in 10 mL of tap water (pH 8.0; conductivity 200 μS/cm^2^ denoted as TW), as compared to the second via transferring another vial in 10 mL of Na_2_CO_3_ solution (pH 10.0; conductivity of 4008 μS/cm^2^, denoted as AM).

### Electrochemical experiments

Degradation of MC-LR was carried out in a batch system using an undivided cell of 5 mL capacity. The anodes investigated were a carbon cylinder (C), a pure titanium wire (Ti^o^) and synthesized TiO_2_@C. All of them exhibited a cylindrical geometry with an effective geometric average area of 8 cm^2^. The cathode was a platinum wire presenting equivalent area. The exact inter-electrode gap, between anode and cathode was of 0.5 cm.

The applied voltage was controlled by a Tensiometer, consisting in an adjustable DC Power Supply (HF-30035, Hikari, São Paulo, SP, Brazil).

The electrochemical degradation assays were conducted for all anodes and electrochemical batch system using 3 mL of MC-LR sample solutions, TW and AM, in each assay.

Experiments were carried out at the applied voltages of 1.5, 2.5 and 5.0 V for 10, 30 and 60 minutes. MC-LR degradation was monitored by UV-Vis spectrometry and differential pulse voltammetry (DPV).

The spectrophotometric measurements were conducted in a UV-Visible Spectrophotometer (Quimis Aparelhos Científicos, model Q798U2VS) coupled to Unico Application Software (S2100 Series UV/Vis). Spectra were scanned from 190 to 800 nm. DPV measurements were performed with a potentiostat/galvanostat μAutolab III^®^ integrated to the GPES 4.9^®^ software, Eco-Chemie, Utrecht, The Netherlands. Measurements were performed in a 5.0 mL one-compartment electrochemical cell, with a three-electrode system consisting of a glassy carbon electrode, a Pt wire and the Ag/AgCl/KCl 3 M (both purchased from Lab solutions, São Paulo, Brazil), representing the working electrode, the counter electrode and the reference electrode, respectively. The experimental conditions for DPV were: pulse amplitude 50 mV, pulse width 0.5 s and scan rate 10 mV s^−1^, all performed at room temperature without pH and conductivity control.

The electro-oxidation assessment was performed regarding the electrochemical efficiency (EE) using equation (2).


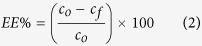


where, C_0_ is the initial concentration and C_f_ is the final concentration.

### MS analysis of MC-LR degradation products

Mass spectrometry analysis was carried out in a mass spectrometer microTOF III (Brucker Daltonics, Bremen, Germany) equipped with a commercial ESI (Brucker Daltonics, Bremen, Germany) source set to operate over *m/z* 150–1100. Samples were extracted using dichloromethane and then methanol-diluted to a (1:1) ratio, followed by acidification with 0.2% formic acid. The resulting solution was directly injected with a flow rate of 5 μL.min^−1^, all analyses were performed in the positive mode. ESI( + ) source conditions were as follows: nebulizer nitrogen gas temperature and pressure of 2.0 bar and 200 °C, capillary voltage of −4 kV, transfer capillary temperature of 180 °C; drying gas of 4 L.min^−1^; end plate offset of −500 V; skimmer of 35 V and collision voltage of −1.5 V. Each spectrum was acquired using 2 microscans. The resolving power (*m*/Δ*m*_50%_ 16.500,00, where Δ*m*_50%_ is the peak full width at half-maximum peak height). Mass spectra were acquired and processed with Data Analysis software (Brucker Daltonics, Bremen, Germany).

## Additional Information

**How to cite this article**: Lobón, G. S. *et al*. Efficient electrochemical remediation of microcystin-LR in tap water using designer TiO_2_@carbon electrodes. *Sci. Rep.* **7**, 41326; doi: 10.1038/srep41326 (2017).

**Publisher's note:** Springer Nature remains neutral with regard to jurisdictional claims in published maps and institutional affiliations.

## Figures and Tables

**Figure 1:**
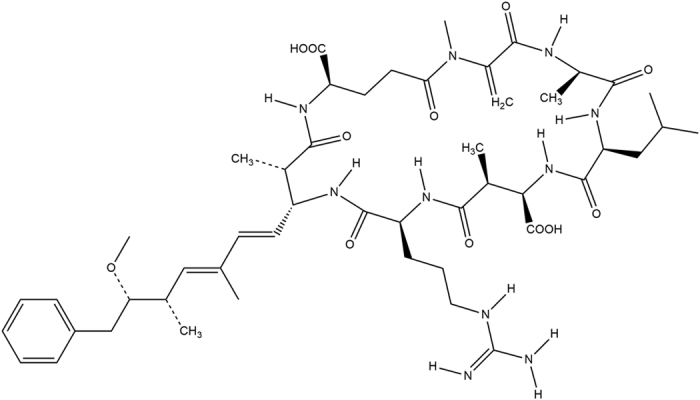
Reported structure of Microcystin-LR.

**Table 1:** Microcystin-LR degradation products identified by ESI(+) MS/MS.

*m/z*	Composition and sequence
218.2125 (I)	Leu-MeAsp – (NH_2_) Or Adda – (−134 Adda) + (2 OH)
274.2769 (II)	Arg-MeAsp – (NH) + (O)
302.3074 (III)	Glu-Mdha – (CH_2_) + (O)
318.2028 (IV)	Arg-MeAsp + (2 O)
540.5383 (V)	Arg-MeAsp-Leu-Ala-Mdha-Glu
851.3981 (VI)	*Cyclo*[Adda-Glu-Mdha-Ala-Leu-MeAsp-Arg-Adda] – (177Adda) + (O) + (OH)
